# A novel natural killer-related signature to effectively predict prognosis in hepatocellular carcinoma

**DOI:** 10.1186/s12920-023-01638-0

**Published:** 2023-09-06

**Authors:** Deyang Xi, Jialu Wang, Yinshuang Yang, Fang Ji, Chunyang Li, Xuebing Yan

**Affiliations:** 1 Department of Infectious Diseases, Affiliated Hospital of Xuzhou Medical University, Xuzhou, Jiangsu Province China; 2 Department of Radiology, Affiliated Hospital of Xuzhou Medical University, Xuzhou, Jiangsu Province China

**Keywords:** Natural killer cell, HCC, Tumor microenvironment, Prognostic signature

## Abstract

**Background:**

Hepatocellular carcinoma (HCC) is a prevalent tumor that poses a significant threat to human health, with 80% of cases being primary HCC. At present, Early diagnosis and predict prognosis of HCC is challenging and the it is characterized by a high degree of invasiveness, both of which negatively impact patient prognosis. Natural killer cells (NK) play an important role in the development, diagnosis and prognosis of malignant tumors. The potential of NK cell-related genes for evaluating the prognosis of patients with hepatocellular carcinoma remains unexplored. This study aims to address this gap by investigating the association between NK cell-related genes and the prognosis of HCC patients, with the goal of developing a reliable model that can provide novel insights into evaluating the immunotherapy response and prognosis of these patients. This work has the potential to significantly advance our understanding of the complex interplay between immune cells and tumors, and may ultimately lead to improved clinical outcomes for HCC patients.

**Methods:**

For this study, we employed transcriptome expression data from the hepatocellular carcinoma cancer genome map (TCGA-LIHC) to develop a model consisting of NK cell-related genes. To construct the NK cell-related signature (NKRLSig), we utilized a combination of univariate COX regression, Area Under Curve (AUC) LASSO COX regression, and multivariate COX regression. To validate the model, we conducted external validation using the GSE14520 cohort.

**Results:**

We developed a prognostic model based on 5-NKRLSig (IL18RAP, CHP1, VAMP2, PIC3R1, PRKCD), which divided patients into high- and low-risk groups based on their risk score. The high-risk group was associated with a poor prognosis, and the risk score had good predictive ability across all clinical subgroups. The risk score and stage were found to be independent prognostic indicators for HCC patients when clinical factors were taken into account. We further created a nomogram incorporating the 5-NKRLSig and clinicopathological characteristics, which revealed that patients in the low-risk group had a better prognosis. Moreover, our analysis of immunotherapy and chemotherapy response indicated that patients in the low-risk group were more responsive to immunotherapy.

**Conclusion:**

The model that we developed not only sheds light on the regulatory mechanism of NK cell-related genes in HCC, but also has the potential to advance our understanding of immunotherapy for HCC. With its strong predictive capacity, our model may prove useful in evaluating the prognosis of patients and guiding clinical decision-making for HCC patients.

**Supplementary Information:**

The online version contains supplementary material available at 10.1186/s12920-023-01638-0.

## Background

Hepatocellular carcinoma represents the most prevalent form of malignant liver tumors in adults. Recent global cancer statistics indicate that primary liver cancer ranks as the sixth most frequently diagnosed cancerous condition, while simultaneously holding the second highest mortality rate [1]. At present, surgical resection and liver transplantation are considered the primary treatment options for liver cancer. However, the stealthy onset and rapid progression of liver cancer often result in late-stage diagnoses and metastases, making it challenging to treat [2]. Later stage diagnosis and metastasis often accompany liver cancer in many patients [3]. Despite the promising progress and widespread use of targeted therapy [4], the overall survival rate of patients with HCC has remained largely unchanged due to drug resistance and adverse effects associated with this treatment modality. Therefore, there is an urgent need to study new treatment targets and biomarkers to better improve the prognosis of patients. NK cells are important immune cells in the body [5]. Compared to B cells and T cells, NK cells have the unique ability to kill tumor cells in a nonspecific manner, without requiring prior sensitization [6]. NK cells play a crucial role in the immune response against tumors by effectively eliminating cancer cells [7]. Furthermore, NK cells can also play an important indirect immune-mediated role by cooperating with other innate immune cells to regulate the immune status and function of the body. They secrete various chemokines and cytokines that enhance immune defense and help to maintain the immune balance of the body [8]. Therefore, it is considered a potential target for cancer immunotherapy [9]. In recent years, immunotherapy, as the fourth treatment after surgery, radiotherapy, and chemotherapy, has been gradually applied to clinical practice [10]. In addition to the focus on chimeric antigen receptor T-cell (CRT-T) therapy, NK cell-based immunotherapy has emerged as a novel approach for treating both solid and hematologic tumors [11]. It has been reported that NK cell immunotherapy has greatly improved the adverse reactions and survival time of patients with liver cancer, which can effectively improve the quality of life of patients [12]. Currently, the significance of NK cell-related genes in the prognosis of HCC patients is not well understood. Thus, this study aims to conduct a comprehensive analysis of the relationship between the expression of genes associated with NK cells and the prognosis of HCC patients.

## Materials and methods

### Dataset and sample extraction

RNA sequencing data (RNA-seq), clinical features, and mutation data of TCGA-LIHC cohort dataset were obtained from The Cancer Genome Atlas (TCGA, https://portal.gdc.cancer.gov/) [13]. To begin with, data from 424 HCC patients were initially collected from the TCGA database. Patients with incomplete follow-up data or a survival time of less than 30 days, as well as those lacking complete clinical data, were excluded from the follow-up analysis. Ultimately, a total of 319 cancer patients and 50 healthy controls were enrolled. To account for the same genes with different probes, the average was taken. The data was then standardized using log2 (TPM + 1) transformation, and was normalized by “normalizeBetweenArrays” function of "limma" R package. To obtain the necessary data for this study, we downloaded the GSE14520 dataset from the Gene Expression Omnibus (GEO) database (https://www.ncbi.nlm.nih.gov/geo/) [14]. We carefully excluded patients who had a survival time of less than 30 days, lacked clinical data, or had incomplete follow-up data. In total, 177 cases of HCC patients were included in the study. And standardize the data with the "limma" R package. GSE14520 is considered an externally validated dataset. To investigate the relationship between NK-cell-related genes and the prognosis of HCC patients, a total of 273 NK-cell-related genes were collected. These genes were identified by gathering 134 NK-cell-related genes from the ImmPort Portal website (Additional file 1. Table S1) and 31 gene sets obtained from the MSigDB database (Additional file 2. Table S2). Finally, we removed the duplicate genes, resulting in a final list of 273 NK-cell-related genes. This study is based on the Helsinki Declaration, which was revised in 2013.

### Identification of NKRLSig and formulation of modal for prediction of prognosis of HCC patients

To investigate the differential expression of NK cell-related genes between normal and tumor tissues, we employed the "limma" R package, a widely used tool for analyzing microarray and RNA-seq data. The screening criterion we used was a threshold of |log2FC|> 0.585 and an adjusted *p*-value of less than 0.05 [15]. To construct the 5-NKRLSig, we intersected the differentially expressed genes obtained from the analysis of normal and tumor tissues with known NK cell-related genes. This approach allowed us to identify a list of genes that were differentially expressed and also functionally related to NK cells. Through univariate COX regression with *p* < 0.05 [16]. We identified the NK regulatory genes related to survival. Then we used "survivalROC" R package to calculate AUC [17] and "glmnet" package to run LASSO COX regression analysis [18] in the training set. To further screen genes, we utilized a five-fold cross-validation method to determine the penalty regularization parameters. Finally, to determine the most significant genes for the 5-NKRLSig model, we used multivariate COX regression analysis to identify the central gene and its coefficients. Based on the central gene and coefficients, we constructed the 5-NKRLSig model and calculated the risk score using the regression coefficient obtained from the multivariate COX regression analysis. The risk scoring formula is established as follows: Risk score = ExpressionmRNA1 × CoefmRNA1 + ExpressionmRNA2 × CoefmRNA2 + ExpressionmRNA3 × CoefmRNA3 + ExpressionmRNA4 × CoefmRNA4 + ExpressionmRNA5 × CoefmRNA5. According to the risk score, the cut-off point was calculated using the ‘surv.cutpoint’ function of "survminer" R package based on the minimum P-value. We divided all patients in the TCGA-LIHC cohort into high- and low-risk groups based on the cut-off value and plotted their respective Kaplan–Meier survival curves [19]. Finally, the accuracy of the model was evaluated using Time-ROC analysis [20].

### Functional enrichment analysis

Gene Ontology (GO) is a structured standard biological model that covers three aspects: cell composition (CC), molecular function (MF), and biological process (BP) [21]. Kyoto Encyclopedia of Genes and Genomes (KEGG) is a database that provides a systematic and comprehensive mapping of genes and expression information to pathways, which aids in the understanding of gene metabolic processes in organisms. We utilized the "clusterProfiler" R package to perform GO and KEGG enrichment analysis of 115 NK-related genes, with a significance threshold of *p* < 0.05 [22]. The observed differences were found to be statistically significant, and to facilitate visualization of the results, we used the "circlize" R package. In addition, Gene set enrichment analysis (GSEA) was conducted to identify distinct biological processes and signaling pathways that differentiate high-risk and low-risk groups in HCC.

### Construction of the nomogram

We integrated the established NKRLSig model with clinical information to evaluate the independent prognostic value of the risk score, using univariate COX regression, LASSO COX regression and multivariate COX regression. To improve clinical applicability, we further developed a nomogram by integrating the genetic model and clinical information, and created it using the "rms" R package. The nomogram was used to predict the 1-, 3-, and 5-year survival status of patients in the TCGA-LIHC cohort [23].

### Analysis of somatic mutation data and TMB

TMB, which stands for tumor mutational burden, represents the sum of base substitution, insertion, and deletion mutations per trillion bases in the tumor exon coding region [24]. To visually analyze the number of copy number variations of somatic non-synonymous mutations in each sample, we utilized the "maftools" R package [25]. Additionally, we compared the TMB of the high- and low-risk groups and generated abrupt OncoPrint diagrams using the "ComplexHeatmap" R package [26].

### Tumor microenvironment analysis

The "Estimate" R package was utilized to estimate the composition of the immune matrix in the HCC tumor microenvironment (TME) [27]. Subsequently, the immune score, matrix score, and ESTIMATE score were compared between the low-risk and high-risk groups.

### Evaluation of immunotherapy, chemotherapy and target therapy based on risk score

To further evaluate the clinical utility of the risk score, we utilized the TIDE algorithm [28] to predict the immunotherapy sensitivity of the high-risk and low-risk groups. Additionally, we assessed the immune predictive score (IPS) of the two groups to predict the response to immunotherapy [29]. We obtained the patient immunotherapy data from the Cancer Immunotherapy Response Prediction and Outcome (CRIPO) group database and analyzed the response of patients in the high-risk and low-risk groups to different chemotherapeutic drugs using the "pRRophetic" R package to predict the semi-inhibitory concentration (IC50) [30].

### Statistical analysis

The data were analyzed using R software version 4.2.1. Univariate COX regression analysis, AUC, LASSO-COX regression analysis, and multivariate COX regression analysis were employed to construct the 5-NKRLSig model. The Kaplan–Meier (KM) survival curve was used to compare the overall survival (OS) of the high-risk and low-risk groups. The performance of the model was evaluated using a time-dependent receiver operating characteristic (ROC) curve. The Wilcoxon rank-sum test was utilized to compare the proportion of tumor infiltrating immune cells, immune checkpoints, and immune function between the two groups. *P* value of less than 0.05 was considered statistically significant.

## Results and discussion

### Screening of NK cell-related genes and construction of the NKRLSig model

**Fig. 1:**
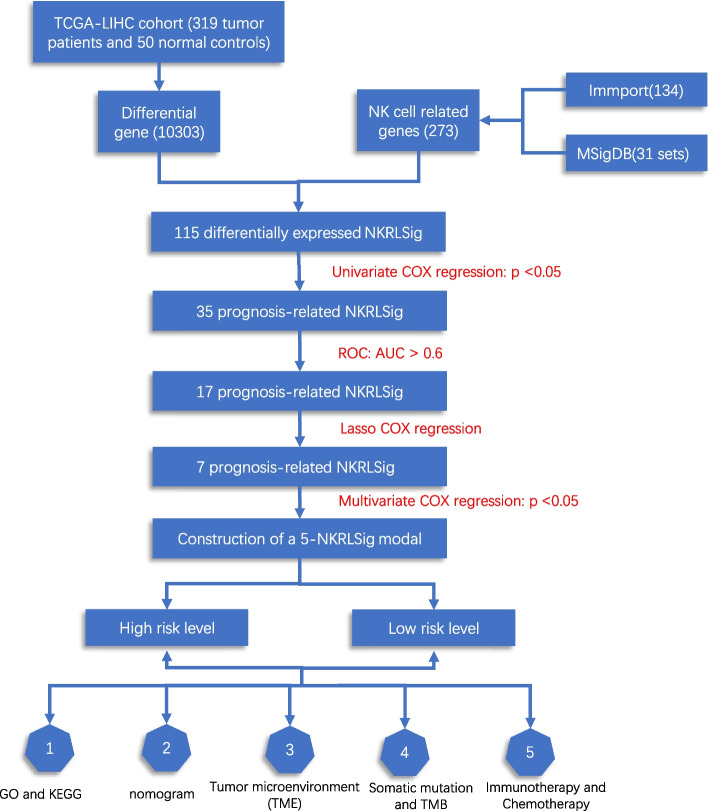
Working flow chart, TCGA-LIHC: The Cancer Genome Atlas-Live Hepatocellular Carcinoma, ROC: Receiver operating characteristic

**Fig. 2:**
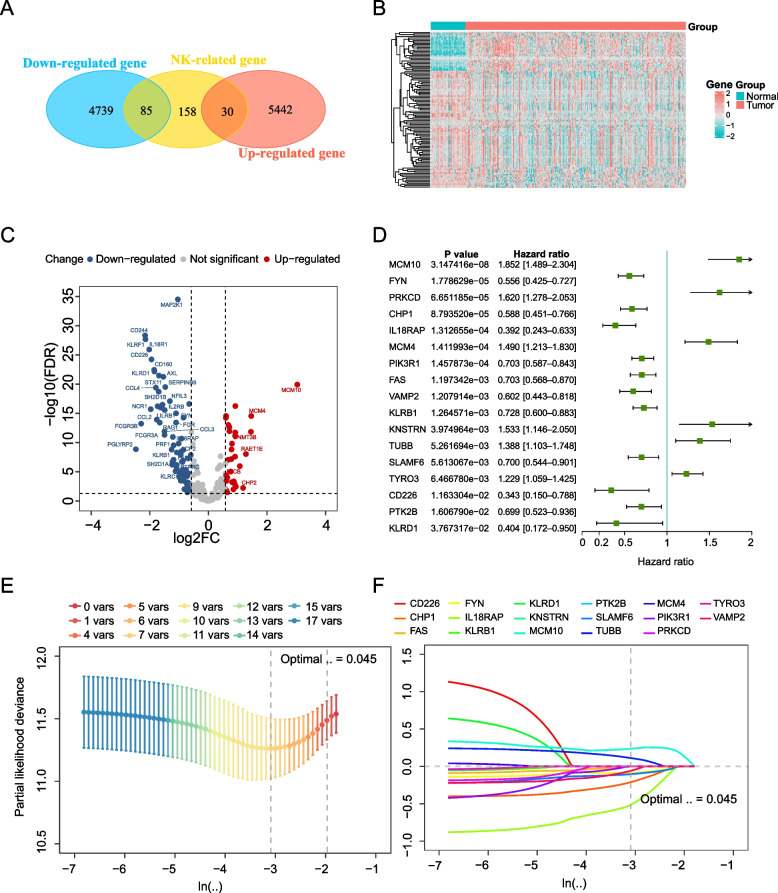
Construction of the NKRLSig model based on the target genes **A** A Venn diagram depicts 115 overlapping mRNAs considered NK cell-related, including 85 down-regulated genes and 30 up-regulated genes. **B** Heat map plot differentially expressed NKRLSig. **C** Volcano plot differentially expressed NKRLSig. **D** Forest plot showing 17 mRNAs with hazard ratios (95%confidence intervals) and P values based on the result of univariate Cox regression analysis. **E–F** mRNAs screened by the LASSO-Cox regression model

Figure 1 outlines the main design of this study. We obtained the intersection of NK cell genes, and a total of 85 up-regulated genes and 30 down-regulated genes were obtained (Fig. 2A). Next, we utilized the "pheatmap" R package to perform a comprehensive visual analysis, generating both a heat map (Fig. 2B) and a volcano map (Fig. 2C) to represent the differential expression of genes related to NK cells. Furthermore, the results of univariate COX regression analysis revealed that a total of 35 NK cell-related genes were significantly associated with the prognosis of HCC patients. The corresponding p-values and hazard ratios (HR) were also presented (Additional file 3. Table S3). To improve the model's accuracy, we performed single-factor screening and calculated the AUC for each gene [17]. Genes with an AUC value greater than 0. 6 were used as our screening condition. The ROC curve is significant because an AUC value greater than 0.6 indicates high precision in predicting patient survival using the gene. Figure 2D shows the P and HR values of variables filtered by univariate COX regression and AUC (Fig. 2D). To improve variable screening, we employed LASSO COX regression and conducted fivefold cross-validation to obtain the λ value, which was determined as the lowest point on the cross-validation plot. Subsequently, we screened the variables and generated a LASSO regression curve (Fig. 2E) and a cross-validation plot (Fig. 2F). Seven genes selected by LASSO regression (IL18RAP, CHP1, MCM10, PRKCD, PIK3R1, VAMP2, FYN) were included in multivariate cox regression and the coefficient was calculated.

**Fig. 3:**
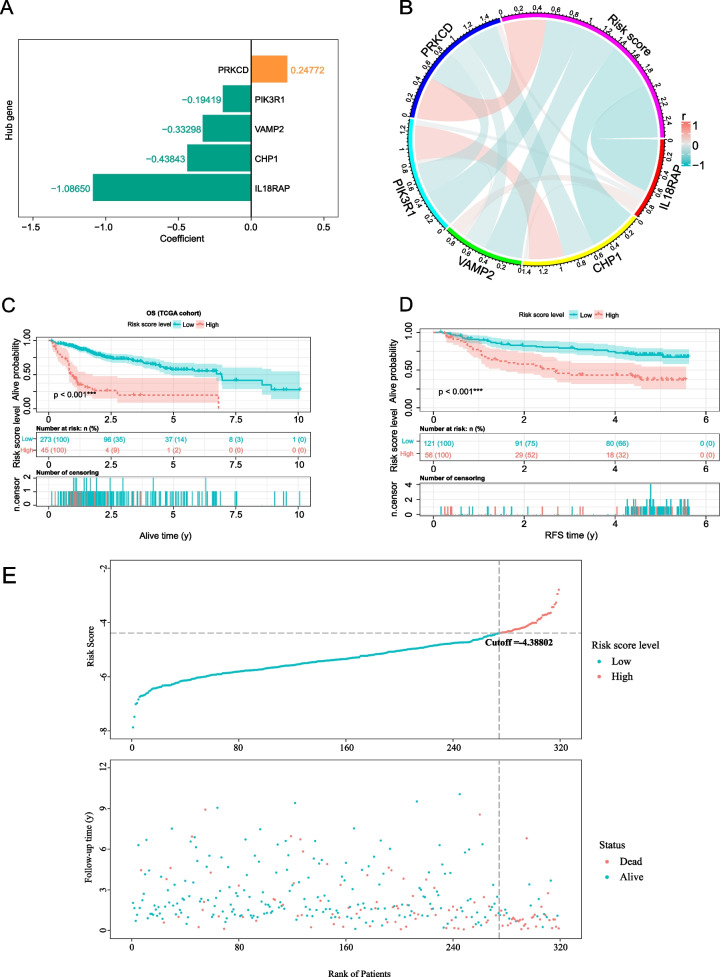
Evaluation of the predictive efficacy of the prognostic model. **A** The multivariate Cox relapse coefficient. **B** Circus plot show the correlation between risk scores and target genes. Kaplan–Meier survival curves in the high- and low-risk groups stratified by risk scores for overall survival in the training set **C** and test set **D**. **E** The risk score distribution and patient status for the TCGA-LIHC cohort

According to the results of the multivariate COX regression (Fig. 3A), we established the risk score formula: risk score = (- 1.08665 × IL18RAPexpression) + (- 0.43843 × CHP1expression) + (- 0.33298 × VAMP2Gexpression) + (- 0.19419 × PIK3R1expression) + (- 0.24772 × PRKCDexpression). To provide a more visual representation of the relationship between the target gene and the risk score, we constructed a correlation circus plot for the risk scores (Fig. 3B). According to the risk score, the cut-off point value was calculated to be -4.3882 by using "survminer" R package “sur.cutpoint” function, and all patients in the TCGA-LIHC cohort were divided into high-risk and low-risk groups. Finally, the study cohort consisted of 319 HCC patients, with 45 individuals in the high-risk training group and 274 in the low-risk training group. In the high-risk testing group, there were 56 patients, while in the low-risk testing group, there were 121 patients. Survival analysis was conducted separately on the training set and validation set, and the Kaplan–Meier analysis results (Fig. 3C-D) demonstrated that patients with low risk had a better prognosis compared to those with high risk. Furthermore, we generated a risk score distribution map (Fig. 3E) to visualize the association between risk scores and patient mortality, revealing that risk scores were significantly elevated in patients who had experienced more deaths. Additionally, differential expression analysis revealed distinct expression profiles of the five mRNAs between the high and low-risk groups, underscoring the close relationship between these mRNAs and patient prognosis in our study.

### Evaluation of the precision of the 5-NKRLSig model

**Fig. 4:**
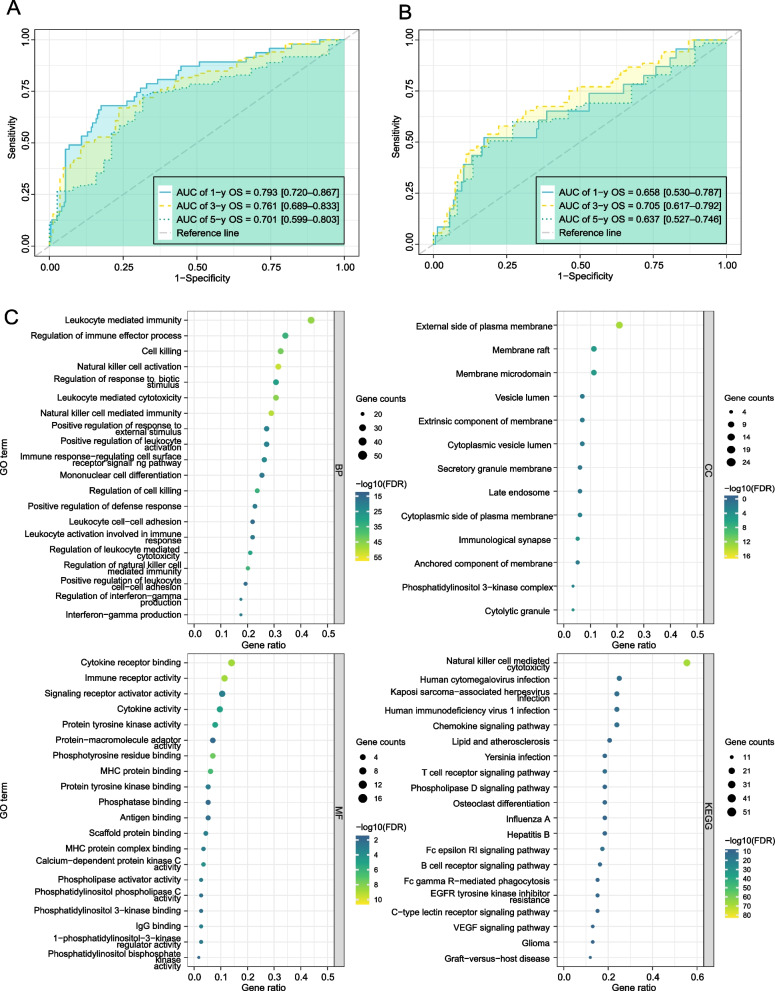
Verified the accuracy of the prognostic model. Time-dependent ROC curves analysis in the train set **A** and test set **B**. **C** GO and KEGG functional enrichment analysis of NKRLSig

To further evaluate the accuracy of the 5-NKRLSig model we constructed, we drew time-dependent ROC curves for the training and testing sets (Fig. 4A-B). The AUC results further demonstrated the model's accuracy. Specifically, the AUC for the first, third, and fifth year in the training set were 0.793 (0.720–0.867), 0.761 (0.689–0.833), and 0.701 (0.599–0.803), respectively. In the external testing set (GSE14520), the AUC for the 1st, 3rd, and 5th years were 0.658 (0.530–0.787), 0.705 (0.617–0.792), and 0.637 (0.527–0.746), respectively. Overall, our model demonstrated good prediction ability for the accuracy of 1, 3, and 5 years in both the training and testing sets.

### Functional enrichment analysis

We conducted a comprehensive analysis of the differential expression of genes related to NK cells using GO and KEGG enrichment analysis. The selection criteria for significantly enriched items were a threshold of FDR < 0.05 and *P* < 0.05. GO enrichment analysis categorized the differential genes into molecular biological function, biological process, and cellular components to gain a more comprehensive understanding of gene function. The KEGG pathway enrichment score was used to functionally annotate differentially expressed genes to understand the related functions and pathways of these genes. In addition, we also used GSEA enrichment analysis between high- and low-risk groups.

Our analysis of 115 differential genes related to NK cells revealed that the biological processes with higher abundance were leukocyte-mediated immunity, regulation of immune effector processes, and cell killing. The most abundant cellular components were the external side of the plasma membrane, membrane raft, and membrane microdomain. The most abundant biological functions were cytokine receptor binding, immune receptor activity, and signaling receptor activator activity. In terms of KEGG pathway enrichment, the pathways with higher abundance were natural killer cell-mediated cytotoxicity, human cytomegalovirus infection, and Kaposi sarcoma-associated herpesvirus infection (Fig. 4C).

**Fig. 5:**
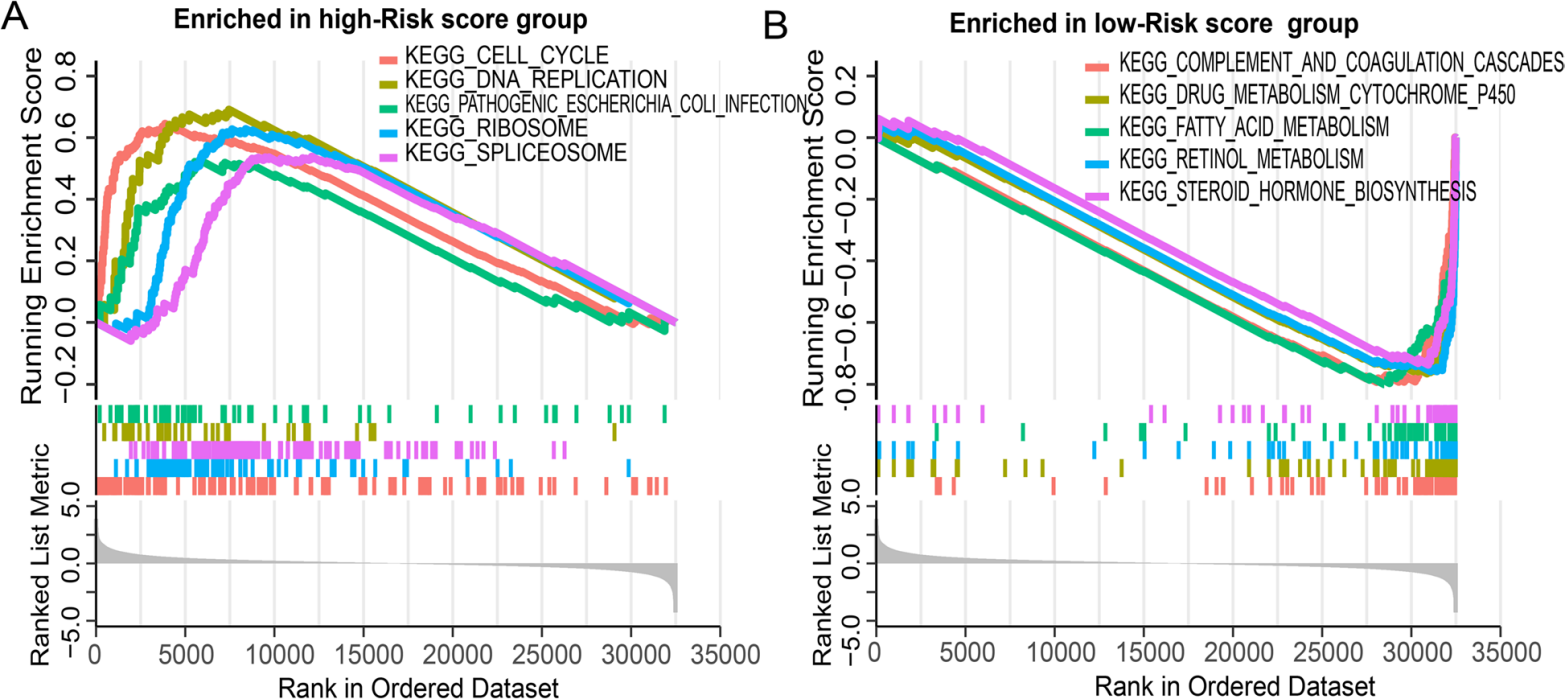
GSEA enrichment analysis identifies KEGG pathways associated with high-risk **A** and low-risk groups **B** in the training set

In this study, we performed Gene Set Enrichment Analysis (GSEA) to compare the high-risk and low-risk groups in the training set. The results revealed distinct enrichment patterns in each group. Specifically, the high-risk group showed significant enrichment in pathways associated with cell cycle, DNA replication, pathogenic Escherichia coli infection, ribosome, and spliceosome (Fig. 5A). Conversely, the low-risk group exhibited enrichment in pathways related to complement and coagulation cascades, drug metabolism cytochrome P450, fatty acid metabolism, retinol metabolism, and steroid hormone biosynthesis (Fig. 5B). These findings provide valuable insights into the underlying molecular mechanisms that contribute to the differential prognostic outcomes observed between the high-risk and low-risk groups in our study.

### Identifying the role of risk level in clinical subgroups

**Fig. 6:**
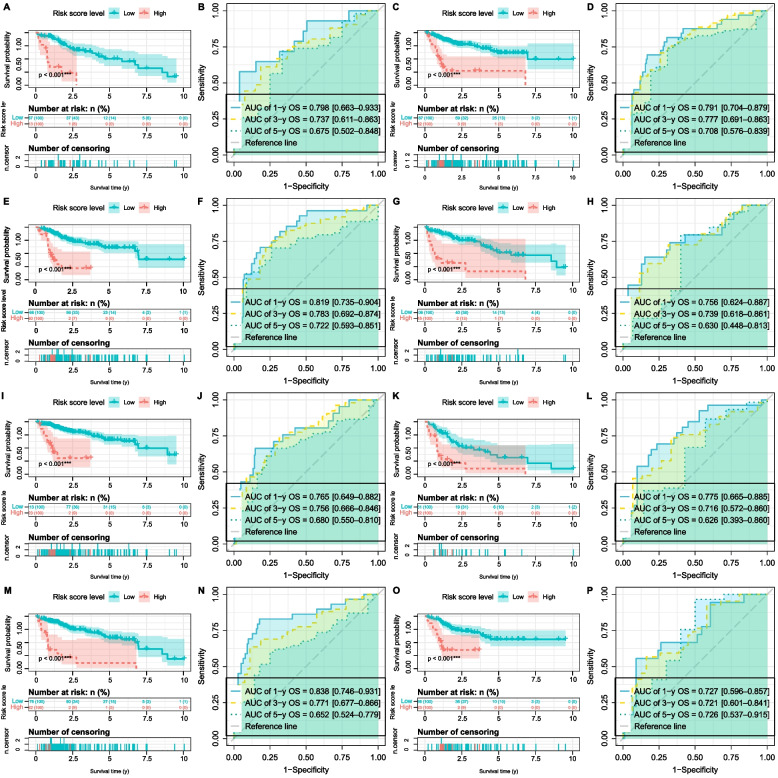
Clinical application of the 5-NKRLSig model in HCC. The difference in risk score by Female **A-B**, Male **C-D**, Age < 65 **E–F**, Age ≥ 65 **G-H**, Stage I/II **I-J**, Stage III/IV **K-L**, Grade I/II **M–N**, Grade III/IV **O-P** of HCC

To further investigate whether the prognosis of high-risk and low-risk patients in different clinical subgroups differs, we performed survival analysis on patients in the high and low-risk groups within different clinical subgroups, based on gender (male, female), age (< 65, ≥ 65), stage (stage I/II, stage III/IV), and grade (grade I/II, grade III/IV). The Kaplan–Meier analysis (Fig. 6A-P) indicated that the survival time of low-risk patients was significantly longer than that of high-risk patients. Moreover, time-ROC analysis (Fig. 6A-P) revealed that the constructed 5-NKRLSig model can reliably predict the prognosis of HCC across different clinical subgroups.

### Establishment of nomograms in combination with clinical characteristics

**Fig. 7:**
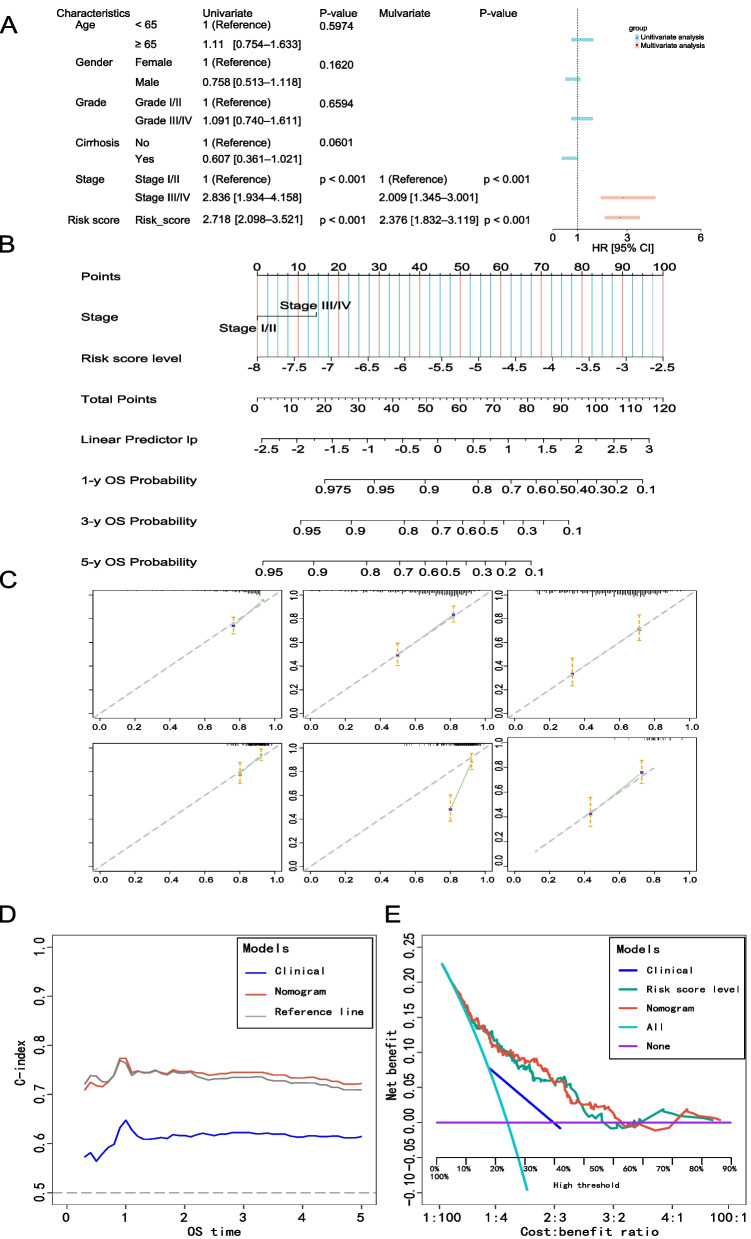
Construction of the nomogram. **A** Univariate and multivariate Cox regression analysis in TCGA-LIHC. **B** Nomogram integrating the Risk score and Stage.** C** Calibration curves for predicting 1, 3and 5 years OS in the train set and test set. **D** Concordance index curves depicting risk scores and other clinical parameters relevant to predicting HCC patient prognosis. **E** DCA of the nomogram and AJCC stage

To enhance the clinical utility of the 5-NKRLSig model developed in this study, we also constructed a nomogram to predict the OS of HCC patients. We conducted univariate and multivariate COX regression analyses on both clinical information and genetic models. According to the results of the multivariate COX regression analysis (Fig. 7A), Stage and risk score were identified as independent influencing factors for HCC. We developed the nomogram (Fig. 7B) based on the patient's stage and risk score, which can more accurately predict the patient's survival rates at 1, 3, and 5 years. The C-index and 95% confidence interval (CI) of the nomogram were 0.743 (0.718–0.769), indicating that the nomogram had good predictive ability for the patient's survival rates at 1, 3, and 5 years (Fig. 7D). Moreover, we drew calibration curves for the training set and testing set respectively (Fig. 7C) to evaluate the consistency between the predicted and actual risks. The calibration curves for both sets demonstrated that the OS probability predicted by the nomogram was in good agreement with the actual OS probability. Furthermore, we compared the performance of our nomogram with AJCC staging using decision curve analysis (Fig. 7E), which revealed that our nomogram was not inferior to AJCC staging in predicting OS of HCC patients.

### Analysis the relationship between 5-NKRLSig modal risk score and somatic mutation and TMB

**Fig. 8:**
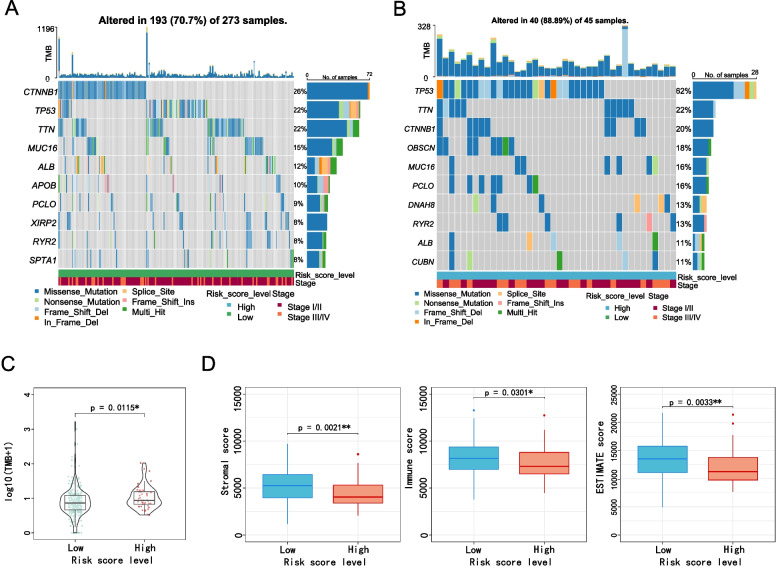
Genetic alterations and tumor microenvironment. Top 10 gene mutations in high-risk **A** and low-risk **B** groups. **C** The analysis of TMB scores of high-risk and high-risk groups. **D** Comparison of the immune score, matrix score and ESTIMATE score in high- and low-risk groups

In this study, we aimed to comprehensively analyze the relationship between the expression of genes related to NK cells and the prognosis of HCC patients. Specifically, we investigated the relationship between the risk score level and somatic mutation and TMB cell mutation. Our findings (Fig. 8A-B) demonstrated that the rate of TP53 somatic mutation was significantly higher in the high-risk group as compared to the low-risk group (62% vs 22%). However, we observed no significant difference in somatic mutation rates of CTNNB1 and TTN between low-risk and high-risk patients. Furthermore, we compared the TMB scores of the high-risk and low-risk groups, and the results (Fig. 8C) showed that high-risk patients had a higher TMB, which was statistically significant.

### Analysis of the tumor microenvironment between high-risk and low-risk groups

The TME constitutes the milieu in which tumor cells thrive and is mainly composed of immune cells and stromal cells. The Estimate algorithm has been developed to estimate the abundance of immune and stromal components in tumors, which can be quantified as an immune score. There have been multiple studies showing that immune and stromal cells play an important role in tumor prognosis. In this study, we employed the "Estimate" R package to calculate the immune score, matrix score, and ESTIMATE score in the high-risk group. Our results revealed that the low-risk group displayed significantly higher immune score, matrix score, and ESTIMATE score compared to the high-risk group (Fig. 8D).

### Evaluation of immunotherapy, chemotherapy and target therapy based on risk score

**Fig. 9:**
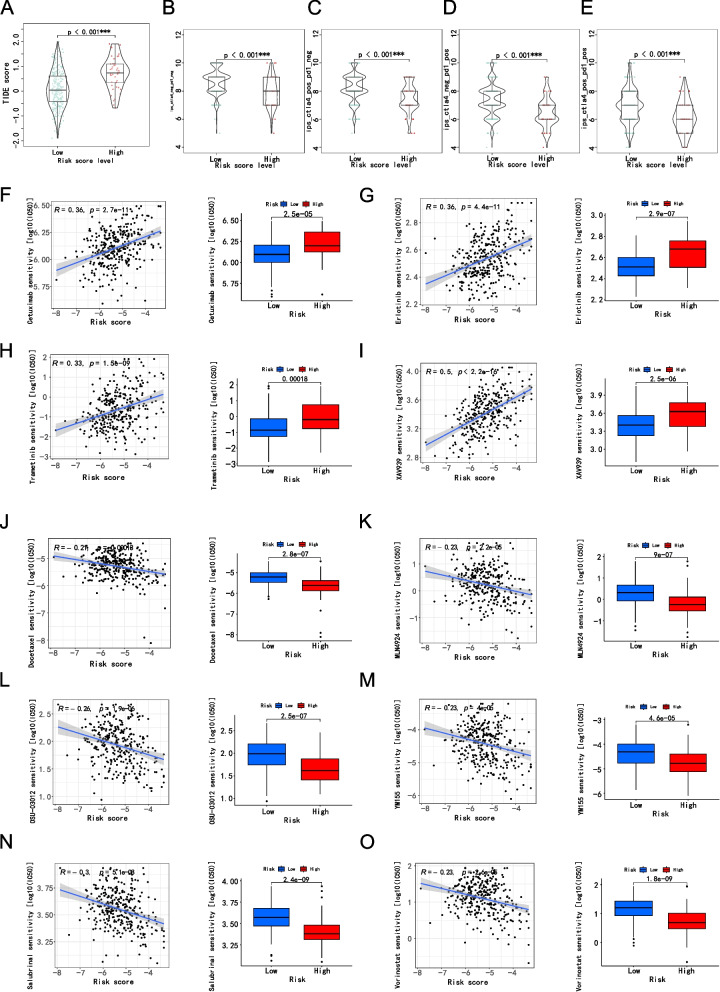
Application of risk score in immunotherapy, Chemotherapy and target therapy. **A** Prediction of immunotherapy response based on the TIDE algorithm. IPS score for immunotherapy. **B** CTLA4- PD1 − . **C** CTLA4 + PD1 − . **D** CTLA4 − PD1 + . **E** CTLA4 + PD1 + . Risk score predicts chemotherapy sensitivity. Cetuximab **F**, Erlotinib** G**, Trametinib **H**, XAV939 **I**, Docetaxel **J**, MLN4924 **K**, OSU-03012 **L**, YM155 **M**, Salubrinal **N**, Vorinostat **O**

Immunotherapy has emerged as a promising treatment option for HCC patients.To further investigate the role of risk score in immunotherapy, we utilized the TIDE algorithm to analyze the response to immunotherapy in two groups. Our results (Fig. 9A) indicate that the low-risk group has a lower TIDE score than the high-risk group, suggesting that the low-risk group is more sensitive to immunotherapy.

Next, we obtained IPS data of HCC patients from the Cancer Immunome Atlas (TCIA) database and analyzed the role of risk score in IPS (Fig. 9B-E). Our results show that the median of the low-risk group was higher than that of the high-risk group, supporting our previous finding that patients in the low-risk group are more sensitive to immunotherapy.

Furthermore, we compared the efficacy of 10 immunotherapeutic drugs in the high-risk and low-risk groups. Our results show that the IC50 of Cetuximab, Erlotinib, Trametinib and XAV939 in the low-risk group was relatively higher (F ig. 9F-I), whereas the IC50 of Docetaxel, MLN4924, OSU-03012, YM155, Salubrinal and Vorinostat in the low-risk group was lower (Fig. 9J-O) compared to the high-risk group. Based on the risk score, we can better understand the immunotherapy of HCC patients and improve the accuracy of drug treatment.

## Discussion

Hepatocellular carcinoma is a highly heterogeneous disease with varying tumor growth patterns and response to treatment. While treatment options such as radiotherapy, chemotherapy, and immunotherapy have provided hope for HCC patients [31], the 5-year survival rate for HCC patients remains less than 20% [32]. One of the main reasons for the poor prognosis of HCC patients is that the disease is often diagnosed at an advanced stage. The conventional risk stratification methods based on tumor size, stage and lymph node metastasis have limited predictive value for the prognosis of patients. Therefore, there is an urgent need for a novel prognostic model. NK cells, with their various cytotoxic mechanisms and immune-regulatory functions through cytokine secretion, have emerged as important players in cancer immunity [33]. However, the regulatory role of NK-related genes in hepatocellular carcinoma has not been reported. Therefore, we established a biomarker prognostic model based on NK-related genes based on TCGA and GEO database. we constructed a 5-NKRLSig and calculated a risk score. We first used the ‘sur.cutpoint’ function to calculate the cutoff point and divide all HCC patients into two prognostic subgroups: high-risk and low-risk groups. Our comprehensive analysis demonstrated that our model has a superior predictive ability when compared with traditional clinical indicators such as age, sex, histological staging, and tumor staging. Importantly, our model also showed good predictive capacity in different clinical subgroups. These findings provide a robust theoretical basis for the clinical application of our model and can significantly improve patient prognosis.

To ensure the accuracy of our model, we employed the method of external testing set (GSE14520) for verification. Despite potential differences in chip specifications, our model's ability to establish a relationship between the risk score and patient prognosis was consistent with results from the training set, suggesting the wide applicability of our approach.

The 5-NKRLSig model constructed in this study comprises IL18RAP, CHP1, VAMP2, PIK3R1, and PRKCD. Previous studies have demonstrated the close association of these five genes with inflammation and tumor occurrence and development. For instance, Wang et al. reported that miR-493-5p overexpression induces apoptosis and inhibits proliferation and migration of hepatocellular carcinoma cells by negatively regulating VAMP expression [34]. Similarly, Ai et al. found that PIK3R1 overexpression activates the PI3K/Akt/mTOR signaling pathway in hepatocellular carcinoma cells, promoting HCC initiation and progression [35]. In the context of natural killer/T-cell lymphoma cells, Lin et al. highlighted the role of IL18RAP in cell growth, where knockout of IL18RAP inhibited NKTCL cell proliferation through cell cycle arrest [36]. CHP1 has been shown to regulate plasma membrane NA( +)/H( +) exchange and play a crucial role in cellular pH regulation [37]. Li et al. reported that lactic acid and low pH can suppress the immune activity of NK cells [38], leading us to speculate that CHP1 may regulate NK cell activity by modulating pH. PRKCD has been identified as an active regulator of mitochondrial autophagy and has significant implications for B cell proliferation and NK cell activation [39]. Ke et al. [40] and Wen J et al. [41] found that PRKCD plays an essential role in the occurrence and development of cancers such as cervical cancer and esophageal squamous cell carcinoma, as well as influencing immunotherapy response. Exploring the application of PRKCD in HCC patients will be a key direction for our future research. These findings underscore the importance of the 5-NKRLSig model and its potential implications in understanding HCC pathogenesis and guiding future therapeutic strategies.

Based on our bioinformatics analysis, the 115 differential genes related to NK cells are primarily enriched in immune regulation and cell killing. We also investigated whether the model gene can be used as a biological marker of prognosis of HCC. Interestingly, the ROC curve analysis demonstrated that the expression of the model gene can accurately predict the survival status of patients, providing theoretical evidence for clinical practice. This study is the first to combine prognostic and diagnostic markers in hepatocellular carcinoma, emphasizing the continuous and inseparable relationship between cancer incidence and prognosis.

Despite the clinical significance of our study in the prognosis evaluation and treatment selection of HCC patients, there are still some limitations that need to be addressed. Our study is retrospective and relies on data from TCGA and GEO databases. Therefore, the applicability of this model as a diagnostic and treatment indicator needs to be further validated in future prospective studies.

## Conclusions

In summary, we have constructed a prognostic model of HCC based on NK-related genes and have verified the effectiveness of the model in an external verification set. This provides new evidence for the evaluation of prognosis and immunotherapy of HCC patients. The study highlights the potential importance of NK cells and their related genes in the prognosis of HCC and may lead to the development of new therapeutic strategies. However, further validation in larger cohorts and prospective studies is needed to confirm the clinical utility of the model.

### Supplementary Information

**Additional file 1: Table S1.** NK-related genes from MSigDB database.

**Additional file 2: Table S2.** NK-related genes from ImmPort Portal website.

**Additional file 3:  Table S3.** Univariate COX regression results and AUC.

## Data Availability

The RNA-seq datasets and clinical datasets are publicly available in the following database: The TCGA-LIHC dataset was downloaded from the GDC portal (https://portal.gdc.cancer.gov/), while the GSE14520 dataset was downloaded from the GEO database (https://www.ncbi.nlm.nih.gov/geo). For further enquiries, please contact the author (Chunyang Li; 13775989791@126.com).
